# Outcomes of preoperative chemoradiotherapy followed by surgery in patients with unresectable locally advanced sigmoid colon cancer

**DOI:** 10.1186/s40880-016-0126-y

**Published:** 2016-07-07

**Authors:** Bo Qiu, Pei-Rong Ding, Ling Cai, Wei-Wei Xiao, Zhi-Fan Zeng, Gong Chen, Zhen-Hai Lu, Li-Ren Li, Xiao-Jun Wu, Rene-Olivier Mirimanoff, Zhi-Zhong Pan, Rui-Hua Xu, Yuan-Hong Gao

**Affiliations:** State Key Laboratory of Oncology in South China, Collaborative Innovation Center for Cancer Medicine, Sun Yat-sen University Cancer Center, Guangzhou, 510060 Guangdong P. R. China; Department of Radiation Oncology, Sun Yat-sen University Cancer Center, No. 651 Dongfeng Road East, Guangzhou, 510060 Guangdong P. R. China; Department of Colorectal Surgery, Sun Yat-sen University Cancer Center, Guangzhou, 510060 Guangdong P. R. China; Department of Radiation Oncology, Centre Hospitalier Universitaire Vaudois, Lausanne, Vaud Switzerland; Department of Medical Oncology, Sun Yat-sen University Cancer Center, No. 651 Dongfeng Road East, Guangzhou, 510060 Guangdong P. R. China

**Keywords:** Unresectable locally advanced sigmoid colon cancer, Neoadjuvant chemoradiotherapy, R0 resection, Down-staging, Organ preservation

## Abstract

**Background:**

Complete resection of locally advanced sigmoid colon cancer (LASCC) is sometimes difficult. Patients with LASCC have a dismal prognosis and poor quality of life, which has encouraged the evaluation of alternative multimodality treatments. This prospective study aimed to assess the feasibility and efficacy of neoadjuvant chemoradiotherapy (neoCRT) followed by surgery as treatment of selected patients with unresectable LASCC.

**Methods:**

We studied the patients with unresectable LASCC who received neoCRT followed by surgery between October 2010 and December 2012. The neoadjuvant regimen consisted of external-beam radiotherapy to 50 Gy and capecitabine-based chemotherapy every 3 weeks. Surgery was scheduled 6–8 weeks after radiotherapy.

**Results:**

Twenty-one patients were included in this study. The median follow-up was 42 months (range, 17–57 months). All patients completed neoCRT and surgery. Resection with microscopically negative margins (R0 resection) was achieved in 20 patients (95.2%). Pathologic complete response was observed in 8 patients (38.1%). Multivisceral resection was necessary in only 7 patients (33.3%). Two patients (9.5%) experienced grade 2 postoperative complications. No patients died within 30 days after surgery. For 18 patients with pathologic M0 (ypM0) disease, the cumulative probability of 3-year local recurrence-free survival, disease-free survival and overall survival was 100.0%, 88.9% and 100.0%, respectively. For all 21 patients, the cumulative probability of 3-year overall survival was 95.2% and bladder function was well preserved.

**Conclusion:**

For patients with unresectable LASCC, preoperative chemoradiotherapy and surgery can be performed safely and may result in an increased survival rate.

## Background

Colorectal cancer is the third most common malignancy and the fourth leading cause of cancer-related death worldwide [1]. In China, it is the fifth most common cancer and the fifth leading cause of cancer death [2, 3]. At initial presentation, approximately 10% of patients with colon cancer have a primary tumor involving adjacent structures, also known as locally advanced disease [4, 5]. Like other gastrointestinal malignancies, sigmoid colon cancer can invade or be adherent to adjacent structures [6–8].

Surgery is the principal treatment of colon cancer and the amount of residual tumor present after surgery is an important predictor of survival and recurrence rate [9]. To achieve resections with microscopically negative margins (R0 resection), treatment of locally advanced adherent colon cancer requires en-bloc multivisceral resection, defined as the partial or complete removal of neighboring organs to which the primary tumor is attached [6, 7]. However, several studies have suggested that many patients with locally advanced colon cancer, even after undergoing multivisceral resection, still have an incomplete removal of tumor [6, 7, 10, 11]. Moreover, this extensive surgical procedure increases the risk of complications and death. These confounding factors have encouraged the evaluation of alternative multimodality treatments.

The efficacy of neoadjuvant chemoradiotherapy (neoCRT) on locally advanced rectal cancer is well established [12–17]; the efficacy of radiotherapy on locally advanced colon cancer, however, is not, with only a few studies having evaluated radiotherapy delivered in an adjuvant manner [18, 19]. Many patients with locally advanced colon cancer undergo extensive surgical resection, or their tumors are deemed unresectable without alternative multimodality approaches being evaluated. We conducted a pilot study to assess the feasibility and efficacy of neoCRT followed by surgery on unresectable locally advanced sigmoid colon cancer (LASCC). The R0 resection rate, pathologic complete response (pCR) rate, organ preservation, treatment complications and survival were analyzed and evaluated.

## Methods

### Patient selection and evaluation

#### Patient eligibility

Patients with LASCC (defined as the primary tumor having an inferior margin >15 cm from the anal verge, as determined by rigid proctoscopy) were candidates for neoCRT if they met the following criteria: (1) curative resection was deemed impossible because preoperative imaging examinations showed that the tumor extensively involved adjacent organs/structures, such as the bladder, ureter, or great vessels, thus compromising a clean radial margin; and/or (2) curative resection was deemed impossible after exploratory laparotomy. Since the primary objective was to assess local tumor response, patients with distant metastasis were also eligible. Patients with uncontrolled medical conditions (e.g., hypertension, diabetes, heart failure, or psychiatric disease) were excluded from the study.

#### Patient evaluation

Patients had to have a computed tomography (CT) scan of the chest and abdomen; magnetic resonance imaging (MRI) of the pelvis; a colonoscopy with/without endoscopic ultrasonography; an electrocardiogram; a complete blood count; a liver function test; a renal function test; test of electrolyte counts; and test of baseline levels of carcinoembryonic antigen (CEA) and carbohydrate antigen 19-9. Patients were staged before neoCRT and after surgery based on the American Joint Committee on Cancer Staging System (7th edition).

### Ethics, consent and permission

This study was approved by the ethics committee of Sun Yat-sen University Cancer Center. Before treatment, written informed consent was obtained from all patients. We discussed the cases of all eligible patients at our Multidisciplinary Cancer Conferences. We obtained consent from all patients to report individual patient data.

### Study design

The primary objective of this prospective study was to assess the R0 resection rate. The secondary objectives were to assess the pCR rate, tumor down-staging, organ preservation, complication rates, local recurrence-free survival (LRFS), disease-free survival (DFS), and overall survival (OS). Organ preservation referred to avoiding extensive multivisceral resection by successful tumor down-staging, thus preserving the structure and function of nearby organs, especially the bladder.

### Treatment

#### Radiotherapy

All patients were immobilized using an AIO Bellyboard and Pelvic Solution system (AIO solution, Orfit Industries, Wijnegem, Belgium). Patients were simulated with moderately full bladder. CT-based simulation with 3-mm slice thickness was performed. The target volumes were delineated according to the guidelines of the International Commission on Radiation Units and Measurements, Reports 50 and 62. The gross tumor volume (GTV) was the macroscopic tumor and the enlarged lymph nodes visible on MRI or CT images. The clinical target volume (CTV) was the primary tumor with a cranio-caudal margin of 2–3 cm, enlarged lymph nodes, sigmoid mesocolon and the lymphatic drainage regions. If the tumor invaded adjacent structures, a further 1.5-cm isotropic margin into the involved structures had to be included to account for microscopic disease and the ischiorectal fossa had to be included to account for possible implantation metastases to the pelvic floor. The cranial boundary of the CTV was located at the interspace between the fourth and fifth lumbar vertebrae. Planning target volume 1 (PTV1) covered the GTV with an isotropic margin of 0.6 cm. PTV2 covered the CTV with an isotropic margin of 0.6 cm. The prescribed dose was 50 Gy to PTV1 and 46 Gy to PTV2 in 25 fractions, with a linear accelerator adjusted to deliver an energy of 8 MV photons. Either 3-dimensional conformal radiotherapy (3D-CRT) or intensity-modulated radiotherapy (IMRT) was delivered. Organ constraints included a maximal dose (D_max_) to the small intestine of less than 54 Gy, a volume of the small intestine receiving >40 Gy (V40 to the small intestine) of less than 150 cm^3^, a volume percentage of the bladder receiving >55 Gy (V55 to the bladder) of less than 30%, a volume percentage of the kidneys receiving >20 Gy (V20 to the kidneys) of less than 30% and a volume percentage of the femoral head receiving >50 Gy (V50 to the femoral head) of less than 5%, whenever applicable.

#### Chemotherapy

Medical oncologists determined the concurrent capecitabine chemotherapy regimen. Adjuvant chemotherapy, consisting of a capecitabine-based regimen, was scheduled for all patients regardless of pathologic tumor, node and metastasis (pTNM) stage.

#### Surgery

MRI of the pelvis, CT of the chest and abdomen, colonoscopy and all blood tests were repeated 5 weeks after neoCRT. Surgery was scheduled 6–8 weeks after radiotherapy. A colectomy with en-bloc removal of the regional lymph nodes was performed. When tumor infiltration or adhesion to the adjacent organs was detected intraoperatively, an en-bloc multivisceral resection was required. En-bloc multivisceral resection was defined as the partial or complete removal of adjacent organs to which the primary tumor was attached, including affected parts of the sigmoid colon.

### Response, toxicity and complications

We defined pCR as the absence, from surgical samples, of malignant cells in the primary site and regional lymph nodes. Acute complications related to neoCRT were evaluated according to the Common Terminology Criteria for Adverse Events (version 4.03). Postoperative complications were evaluated according to the Clavien-Dindo classification [20].

### Follow-up

Patients were followed up every 3 months for the first 2 years after surgery and every 6–12 months thereafter. The last follow-up was performed in October 2015. Surveillance included medical history, physical examination, CEA testing, colonoscopy, and CT scanning. CEA levels were tested and CT scans were performed every 6 months. Colonoscopy was performed every 12 months.

### Statistical analysis

Continuous data are presented as median with range. Categorical data are presented as proportions (%). Cumulative proportions of LRFS, DFS and OS were calculated with Life Table methods. All statistical analyses were performed with SPSS version 17 software (SPSS, Chicago, IL, USA).

## Results

### Demographics

**Fig. 1:**
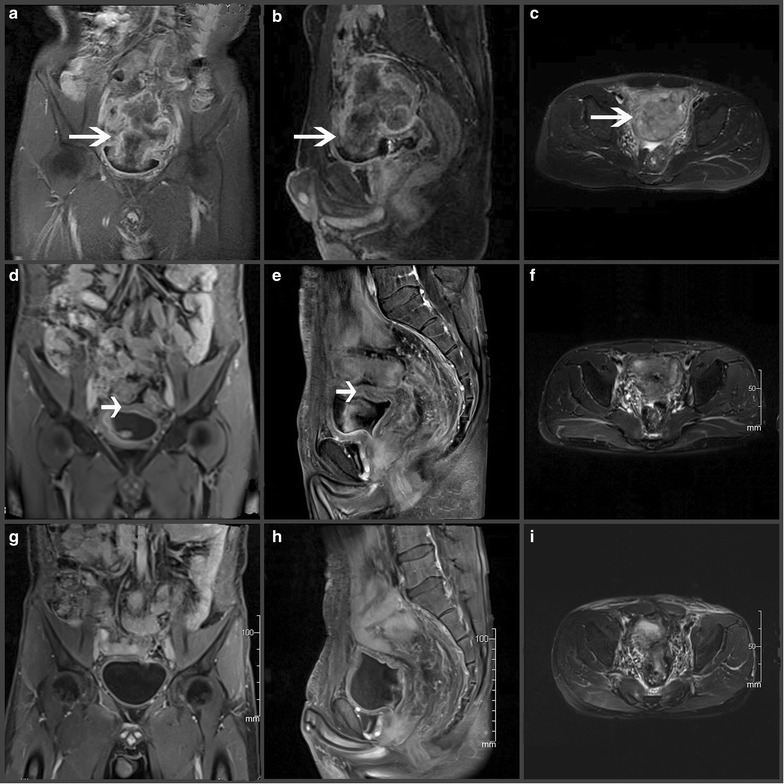
The magnetic resonance imaging (MRI) scans prior to neoadjuvant chemoradiotherapy (neoCRT), after neoCRT and after colectomy in a patient with unresectable locally advanced sigmoid colon cancer (LASCC). MRI scan of the lower abdomen and pelvis before neoCRT (**a**–**c**), 4 weeks after neoCRT (**d**–**f**) and 1 month after surgical resection (**g**–**i**) show the lesion. Prior to neoCRT, the lesion measured 100 mm at the largest dimension and invaded into the urinary bladder (*long arrow*). The radial margins are at risk (**a**–**c**). After neoCRT, substantial down-sizing of the lesion and improvement of all the margins were observed. The upper bladder wall (*short arrow*) remains thick (**d**–**f**). After colectomy and partial cystectomy, the lesion was removed completely. Bladder structure is well preserved (*arrow head*) (**g**–**i**). (**a**, **d**, **g**, coronal T1-weighted with contrast; **b**, **e**, **h**, sagittal T1-weighted with contrast; **c**, **f**, **i**, cross T2-weighted)

In this study, we included 21 patients who were treated at Sun Yat-sen University Cancer Center between October 2010 and December 2012. Patient demographics and tumor characteristics are listed in Table 1. The study population included 16 men and 5 women, with a median age of 47 years (range, 30–74 years). Based on CT/MRI and endoscopic ultrasound results, the baseline primary tumor was classified as T3 in 2 patients, T4a in 7 patients and T4b in 12 patients as well as N0 in 1 patient, N1 in 7 patients and N2 in 13 patients. Involved adjacent structures included the bladder (*n* = 10), ureter (*n* = 4), pelvic/abdominal wall (*n* = 3), uterus (*n* = 2), iliac vessels (*n* = 1) and ileum (*n* = 1), with the bladder being the most frequently involved organ (Fig. 1a–c). Three patients (cases 14, 19 and 21) had hematuria and symptoms of bladder irritation; for these patients, bladder involvement was confirmed by cystoscopy.

**Table 1:** Tumor characteristics and treatment of patients with unresectable locally advanced sigmoid colon cancer

Case	Sex/age (years)	cTNM	Involved structures	Therapy prior to enrollment^a^ (response)	RT (technique, Gy per faction)	Concurrent chemotherapy^a^	Surgery	Resection status	ypTNM	Adjuvant chemotherapy^a^	OS (ms)/status	ECOG PS score
1	F/43	cT4bN2M0	Bladder, pelvic wall	No	3D-CRT, 46/23	XELOX × 2	Colectomy	R0	ypT0N0M0	XELOX × 5	49/FOD	1
2	M/63	cT4bN2M0	Bladder	No	VMAT, 50/25	XELOX × 2	Colectomy	R0	ypT0N0M0	XELOX × 5	36/FOD	1
3	M/59	cT4aN1M0	No	No	VMAT, 50/25	XELOX × 2	Colectomy	R0	ypT0N0M0	XELOX × 1	39/FOD	0
4	M/58	cT3N2M0	No	No	VMAT, 50/25	XELOX × 2 + Xeloda × 1	Colectomy	R0	ypT0N0M0	No	33/FOD	0
5	M/43	cT4aN2M0	No	No	VMAT, 50/25	XELOX × 3	Colectomy	R0	ypT3N0M0	XELOX × 5	30/FOD	1
6	M/50	cT4aN2M1	No	No	VMAT, 50/25	XELOX × 4	Colectomy	R0	ypT3N0M1	FOLFIRI + C225 × 8	28/AWD	0
7	F/67	cT3N2M0	No	No	VMAT, 50/25	XELOX × 2	Colectomy	R0	ypT2N0M0	No	36/FOD	0
8	M/67	cT4bN2M0	Bladder, ureter	No	VMAT, 50/25	XELOX × 2	Colectomy + partial cystectomy + partial ureterectomy	R0	ypT4bN0M0	XELOX × 1	32/FOD	1
9	M/67	cT4aN1M1	No	No	VMAT, 50/25	XELOX × 4	Colectomy	R0	ypT3N0M1	XELOX × 2	17/DOD	–
10	M/70	cT4aN1M1	No	No	VMAT, 50/25	XELOX × 4	Colectomy	R0	ypT3N0M1	No	28/AWD	2
11	M/47	cT4aN1M0	No	No	VMAT, 50/25	XELOX × 4	Colectomy	R0	ypT0N0M0	XELOX × 2	21/FOD	0
12	F/63	cT4bN0M0	Uterus, ureter	No	VMAT, 50/25	XELOX × 3	Colectomy + partial ureterectomy	R0	ypT1N0M0	XELOX × 1	21/FOD	0
13	F/50	cT4aN2M0	No	No	VMAT, 50/25	XELOX × 4	Colectomy	R0	YpT0N0M0	XELOX × 1	21/FOD	0
14	M/30	cT4bN1M0	Bladder	Sigmoidostomy	3D-CRT,46/23	XELOX × 2	Colectomy + partial cystectomy	R0	ypT0N0M0	XELOX × 5	35/FOD	0
15	M/49	cT4bN1M0	Iliac vessels, ureter, ileum	Colostomy	VMAT, 50/25	XELOX × 2 + Xeloda × 1	Colectomy + partial cystectomy + partial ileectomy	R0	ypT3N0M0	XELOX × 2	36/FOD	1
16	F/66	cT4bN2M0	Bladder, uterus	Colostomy	3D-CRT, 46/23	XELOX × 4	Colectomy	R0	ypT3N0M0	No	36/FOD	0
17	M/52	cT4bN2M0	Bladder, ureter	Colostomy	3D-CRT, 46/23	XELOX × 2	Colectomy	R0	ypT3N0M0	XELOX × 4, xeloda × 2	36/FOD	0
18	M/44	cT4bN2M0	Bladder, pelvic wall	Sigmoidostomy	3D-CRT, 46/23	XELOX × 2	Colectomy + biopsy	R2	ypT4aN0M1	No	39/AWD	1
19	M/52	cT4bN2M0	Bladder	Sigmoidostomy, mFOLFOX × 6 (SD)	VMAT, 50/25	CPT11 + Xeloda × 2	Colectomy + partial cystectomy	R0	ypT0N0M0	Xeloda × 1	36/FOD	1
20	M/67	cT4bN1M0	Bladder	Sigmoidostomy, mFOLFOX × 4 (SD), FOLFOXIRI × 3 (SD)	VMAT, 50/25	XELOX × 2	Colectomy + partial cystectomy	R0	ypT3N0M0	–	39/FOD	1
21	M/74	cT4bN2M0	Bladder, abdominal wall	XELOX × 4 (SD)	VMAT, 50/25	XELOX × 2	Colectomy + partial cystectomy	R0	ypT3N0M0	Xeloda × 2	41/FOD	0

*F* female; *M* male; *SD* stable disease; *RT* radiotherapy; *OS* overall survival; *ms* months; *ECOG PS* Eastern Cooperative Oncology Group Performance Status; *FOD* free of disease; *AWD* alive with disease; *DOD* dead of disease; *cTNM* clinical tumor, node and metastasis stage; *ypTNM* pathologic tumor, node and metastasis stage after neoadjuvant therapy. *mFOLFOX* oxaliplatin, 5-fluorouracil and leucovorin; *FOLFOXIRI* 5-fluorouracil, leucovorin, irinotecan and oxaliplatin; *XELOX* capecitabine and oxaliplatin; *CPT11* irinotecan.; *3D*-*CRT* three-dimensional conformal radiotherapy; *VMAT* volumetric modulated arc therapy

^a^The chemotherapy is presented as the regimen × the number of cycles

### Pre-enrollment treatment

Tumor characteristics and treatment of the 21 patients in this study are listed in Table 1. Before being enrolled in this study, 13 patients were treatment-naïve; 7 patients underwent previous surgical exploration without resection of the primary tumor, including diversion of the gastrointestinal tract proximal to the primary tumor in 6 patients and greater omental biopsy in 1 patient; and the remaining 1 patient underwent chemotherapy alone. Three patients received previous chemotherapy regimens that resulted in stable disease: 1 patient received 6 cycles of mFOLFOX (oxaliplatin, 5-fluorouracil and leucovorin); 1 received 4 cycles of mFOLFOX and 3 cycles of FOLFOXIRI (oxaliplatin, 5-fluorouracil, leucovorin and irinotecan); and 1 received 4 cycles of XELOX (capecitabine and oxaliplatin).

### NeoCRT and response

The neoCRT regimen that each patient received is listed in Table 1. Sixteen patients received 50 Gy to PTV1 and 46 Gy to PTV2 in 25 fractions over 35 days. Five patients received 46 Gy to PTV2 without additional boost to PTV1 in order to meet the dose constraints of the small intestine which was adjacent to the primary tumor.

The chemotherapy regimens included 2–4 cycles of XELOX (*n* = 18), 2 cycles of XELOX plus 1 cycle of Xeloda (*n* = 2) and 2 cycles of CPT11 plus Xeloda (*n* = 1).

All patients completed neoCRT within the scheduled time. For all patients, symptomatic improvement was observed and objective response was demonstrated by pelvic MRI after neoCRT (Fig. 1d–f).

### Surgery

All patients underwent surgery according to the protocol. The median interval between radiotherapy and surgery was 8 weeks (range, 7–10 weeks). The median surgery time was 168 min (range, 150–450 min), with a median blood loss of 50 mL (range, 50–200 mL). The median hospital stay after surgery was 8 days (range, 7–19 days). Fourteen patients (66.7%) received a simple colectomy while preserving nearby organs. Multivisceral resection was necessary for the 7 remaining patients (33.3%): 4 patients received colectomy plus partial cystectomy; 1 patient received colectomy plus partial cystectomy and ileectomy; 1 patient received colectomy plus partial cystectomy and partial ureterectomy; and 1 patient received colectomy plus partial ureterectomy. Of the 10 patients with bladder involvement before neoCRT, 6 underwent a partial cystectomy and 4 were able to preserve the whole bladder.

### Adjuvant chemotherapy

The adjuvant chemotherapy regimen was individualized based on previous chemotherapy and response. Among all 21 patients, 13 received capecitabine-based adjuvant chemotherapy, 1 received chemotherapy with FOLFIRI plus cetuximab and 7 refused adjuvant chemotherapy.

### Outcomes

**Fig. 2:**
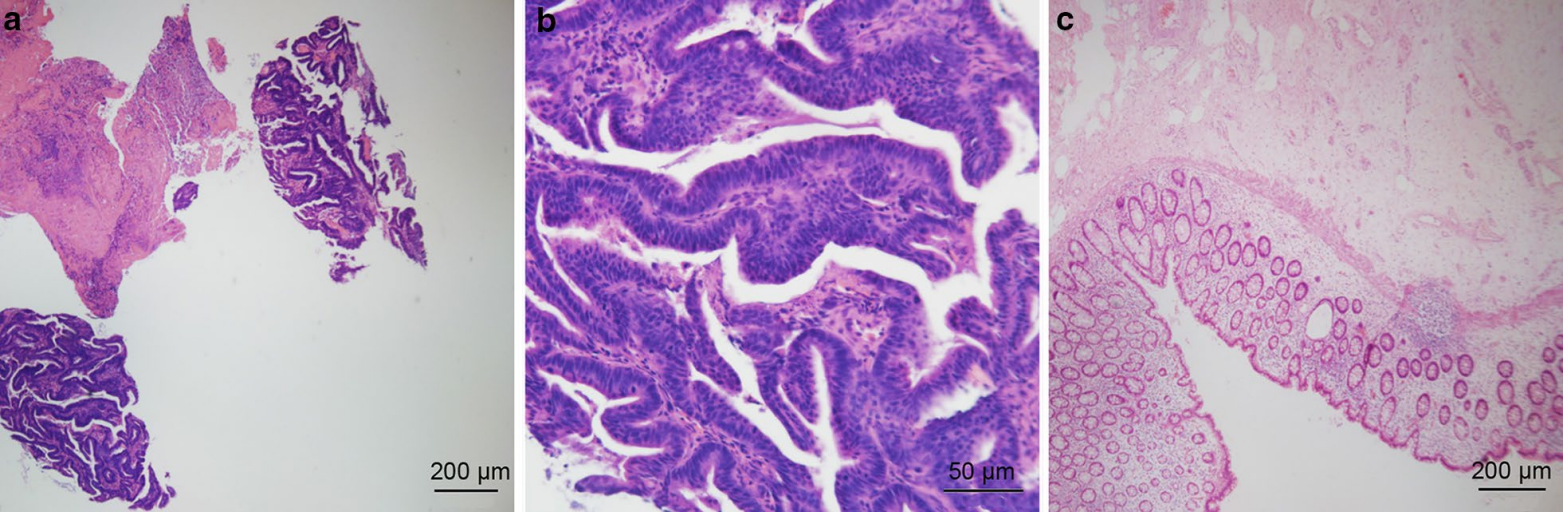
The pathologic findings before neoCRT and after surgery of a patient with LASCC (hematoxylin and eosin staining). **a**, **b** Pathologic examination of the sigmoid colon biopsy before neoCRT shows typical features of a well-differentiated adenocarcinoma: hyperplastic glandular structures lined by atypical epithelial cells. Mitotic figures are observed. **c** Postoperative pathologic examination shows lymphoid infiltrates in intestinal mucosa. No malignant cells are observed

R0 resection was achieved in 20 patients (95.2%). In the remaining 1 patient, tumor deposits on the bladder and pelvic wall were found intraoperatively and the surgeons decided not to remove these deposits. Eight patients (38.1%) had a pCR (Fig. 2). The primary tumor was ypT0 in 8 patients, ypT1 in 1 patient, ypT2 in 1 patient, ypT3 in 9 patients, ypT4a in 1 patient and ypT4b in 1 patient. In all 21 patients, the lymph nodes were negative. The median number of examined nodes was 3 (range, 0–16 nodes). Of the 7 patients who underwent multivisceral resection, only 1 had histologic evidence of tumor invasion to an adjacent organ (the bladder). In the other 6 patients, inflammatory adhesions were found.

**Fig. 3:**
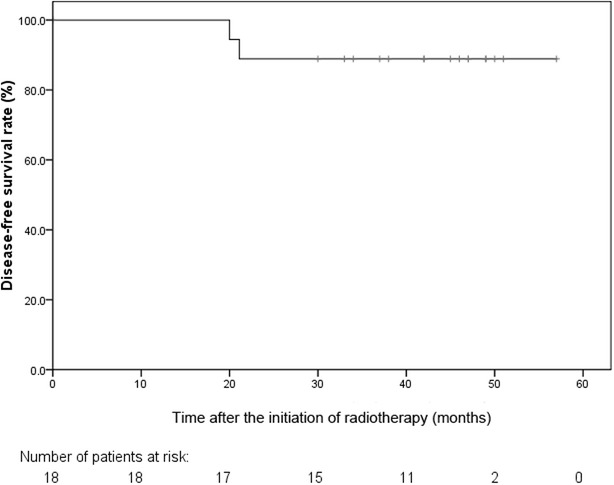
Disease-free survival (DFS) curve of the 18 patients with ypM0 (pathologic M0) sigmoid colon cancer. DFS was calculated by using Life Table methods. The cumulative probability for 3-year DFS rate was 88.9%

At the time of this analysis, the median follow-up time was 42 months (range, 17–57 months). For the 18 patients with ypM0 disease, the cumulative probability for 3-year LRFS, DFS and OS were 100%, 88.9% (95% confidence interval [CI]: 74.4% to 100.0%) and 100%, respectively (Fig. 3). The main failure pattern in the study was distant metastasis (liver metastasis in 1 patient and lung metastasis in another). For all 21 patients, the cumulative probability of 3-year OS was 95.2% (95% CI: 86.2% to 100.0%). At last follow-up, no patient had local failure.

### Treatment-related complications

NeoCRT was generally well tolerated. The most common non-hematologic toxicities observed were gastrointestinal disorders and hand-foot syndrome. Two patients had grade 2 nausea; 3 patients had grade 2 diarrhea; 1 patient had grade 2 hand-foot syndrome; and 1 patient had grade 3 hand-foot syndrome. No grade 4 non-hematologic toxicities were observed.

No patients died within 30 days after surgery. Clavien-Dindo grade 2 postoperative complications were observed in 2 patients (9.5%); of these, 1 patient had pulmonary atelectasis and hypoxia and 1 patient had ileus. Both patients were relieved from these complications after conservative management. No other significant surgical complications, such as impairment of wound healing, anastomotic leakage, fistulas, or infections, were observed.

At last follow-up, 19 patients had an Eastern Cooperative Oncology Group Performance Status score of 0–1; the remaining 2 patients had died of cancer. In all 10 patients with bladder involvement at enrollment, bladder function was well preserved.

## Discussion

The role of neoCRT in the treatment of colon cancer is yet to be determined. In the literature, there are only 3 case reports [21–23] and 4 retrospective studies, each of which enrolled only a small number of patients [24–27]. In the study by Brown et al. [25, 26], neoCRT was delivered to 4 patients with distal sigmoid tumors of T4 disease or threatened circumferential resection margin. Adverse effects were minimal and there was a good tumor down-staging. In another study, Hallet and colleagues [27] focused on recurrent colon cancer. Their results indicated that neoCRT followed by multivisceral resection was a feasible option for selected locally recurrent adherent colon cancer and was able to lead to R0 resection. Cukier et al. [24] reported the largest size of study thus far. Thirty-three patients with primary locally advanced adherent colon cancer received neoCRT followed by multivisceral resection. This approach resulted in high rates of R0 resection and good local control. Our prospective pilot study demonstrated the feasibility of neoCRT in the treatment of patients with unresectable LASCC. In our study, neoCRT achieved an R0 resection rate of 95.2% and a pCR rate of 38.1%; enabled satisfactory organ preservation; and reduced surgery-related morbidity and mortality. We consider our results to be very encouraging.

En-bloc multivisceral resection is the standard surgical procedure for the removal of locally advanced adherent colon tumors [28]. Many studies have evaluated en-bloc multivisceral resection for colon cancer and the complete resection rates range from 40% to 93% [5–10], which for the lowest reported rates is not satisfactory. In our study, prior to neoadjuvant therapy, R0 resection was deemed impossible for all enrolled patients based on imaging or laparotomy findings. In 3 patients, chemotherapy prior to enrollment resulted only in stable disease. NeoCRT improved resectability and the subsequent surgeries achieved an excellent overall R0 resection rate of 95.2%. Similarly, in a study by Cukier et al. [24], 33 patients with primary locally advanced adherent colon cancer received neoCRT followed by multivisceral resection. The R0 resection rate was 100%. Therefore, compared with multivisceral resection alone, neoCRT seems to improve resectability and the R0 resection rate. To our knowledge, residual tumor is a significant negative predictor of survival in patients with locally advanced adherent colon cancer; thus, improvement in the R0 resection rate could result in an improved survival rate.

In our study, neoCRT resulted in significant tumor down-sizing and down-staging. The pCR rate was as high as 38.1% and the pathologic nodal negative rate was 100%. These results could be comparable with those of other colon cancer studies that reported a nodal positive rate of 40% to 69% [8, 29]. However, in their study of locally advanced adherent colon cancer after neoCRT, Cukier et al. [24] reported a pCR rate of 3% and a nodal negative rate of 79%. Both figures are lower than what we found in our study. Higher radiation doses and two-drug combination concurrent chemotherapy might have contributed to this difference. In another study, for the treatment of rectal cancer, neoCRT resulted in high rates of tumor down-staging [30]. In general, patients who experience a pCR after neoCRT have higher DFS and OS rates than those who do not experience a pCR [30, 31]. Therefore, it is reasonable to assume that the final pathologic stage would be an important determinant in sigmoid colon cancer as well, meaning that patients who experience a pCR may have particularly favorable outcomes. However, these conclusions need to be validated by studies that have larger sample sizes and longer duration of follow-up.

Although multivisceral resection is the preferred surgical method for removing locally advanced adherent colorectal tumors, it is an aggressive procedure that increases postoperative morbidity and mortality. The incidence of postoperative complications and mortality were reported to be 11% to 44% and 0% to 7.5%, respectively [7–9]. Therefore, a secondary objective of our study was to evaluate the organ preservation rate after neoCRT. In our study, multivisceral resection after neoCRT was necessary in only 7 of 21 patients and tumor infiltration of an adjacent organ was confirmed histologically in only 1 patient. This suggests that neoCRT can markedly decrease the need for multivisceral resection by sterilizing the peripheral extent of tumor infiltration, which may help to decrease postoperative morbidity and mortality.

According to previous studies as well as to our findings, the urinary bladder is the most frequently involved organ in patients with locally advanced colon cancer [32]. When tumor infiltration of the bladder is suspected, multivisceral resection may include a partial or total cystectomy. This may account for the increased morbidity-related complications, which are associated with bladder repair or urinary diversion [33]. In our study, we observed bladder involvement in 10 patients. After neoCRT, the symptoms related to the involved bladder in these patients were substantially reduced and partial cystectomy was necessary in only 6 patients. None of the patients needed a total cystectomy. In all patients, the urinary structure and function were both well preserved, which substantially improved their quality of life. One example is case 14 (Fig. 1), a 30-year-old man who had gross hematuria and symptoms associated with severe bladder irritation. After the initial evaluation, a total cystectomy as part of multivisceral resection was indicated for this patient. However, after neoCRT, only a partial cystectomy and a colectomy were required. After treatment, this patient had a satisfactory urinary function and was spared from the inconvenience and psychologic pressure that a total cystectomy would cause. Moreover, this patient experienced a pCR, which is indicative of a good prognosis.

3D-CRT or IMRT can accurately deliver radiation to tumors and decrease the dose to abdominal organs, such as the small intestine, liver and kidneys. In this study, chemoradiotherapy was well tolerated. All patients completed the full cycles and doses of chemoradiotherapy and they underwent surgery as scheduled.

We recognize that our study has some limitations. First, the sample size was small and the median follow-up period of 42 months was rather short. Second, the radiation technique/dose and the chemotherapy regimen were not uniform in all patients. Thus, studies that include more patients, have a stricter control of therapy and have a longer follow-up period are required to better determine the efficacy of neoCRT on LASCC.

## Conclusions

For patients with unresectable LASCC, we found that the treatment with neoCRT improved the R0 resection rate, achieved a high pCR rate, enabled satisfactory organ preservation and decreased surgery-related morbidity and mortality. We expect that these advantages might lead to an improvement in survival. To validate our results, studies that enroll more patients and have a longer follow-up period are warranted.

## Abbreviations

CA19-9: carbohydrate antigen 19-9
CEA: carcinoembryonic antigen
CT: computed tomography
CTV: clinical target volume
DFS: disease-free survival
GTV: gross tumor volume
IMRT: intensity-modulated radiotherapy
LASCC: locally advanced sigmoid colon cancer
LRFS: local recurrence-free survival
MRI: magnetic resonance imaging
NeoCRT: neoadjuvant chemoradiotherapy
OS: overall survival
pCR: pathologic complete response
3D-CRT: three-dimensional conformal radiotherapy
